# Coordinate MicroRNA-Mediated Regulation of Protein Complexes in Prostate Cancer

**DOI:** 10.1371/journal.pone.0084261

**Published:** 2013-12-31

**Authors:** Mohammed Alshalalfa, Gary D. Bader, Tarek A. Bismar, Reda Alhajj

**Affiliations:** 1 Department of Computer Science, University of Calgary, Calgary, Alberta, Canada; 2 The Donnelly Centre, University of Toronto, Toronto, Ontario, Canada and the Department of Molecular Genetics, University of Toronto, Toronto, Ontario, Canada; 3 Departments of Pathology, Oncology and Molecular Biology and Biochemistry, Faculty of Medicine, University of Calgary, Alberta, Canada; 4 Biotechnology Research Centre, Palestine Polytechnic University, Hebron, Palestine; University of Pittsburgh, United States of America

## Abstract

MicroRNAs are a class of small non-coding regulatory RNA molecules that regulate mRNAs post-transcriptionally. Recent evidence has shown that miRNAs target entire functionally related proteins such as protein complexes and biological pathways. However, characterizing the influence of miRNAs on genes whose encoded proteins are part of protein complexes has not been studied in the context of disease. We propose an entropy-based framework to identify miRNA-mediated dysregulation of functionally related proteins during prostate cancer progression. The proposed framework uses experimentally verified miRNA-target interactions, functionally related proteins and expression data to identify miRNA-influenced protein complexes in prostate cancer, and identify genes that are dysregulated as a result. The framework constructs correlation matrixes between functionally related proteins and miRNAs that have targets in the complex, and assesses the changes in the Shannon entropy of the modules across different stages of prostate cancer. Results reveal that SMAD4 and HDAC containing protein complexes are highly affected and disrupted by miRNAs, particularly miRNA-1 and miRNA-16. Using biological pathways to define functionally related proteins reveals that NF-kB-, RAS-, and Syndecan-mediated pathways are dysregulated due to miRNA-1- and miRNA-16-mediated regulation. These results suggest that miRNA-1 and miRNA-16 are important master regulators of miRNA-mediated regulation in prostate cancer. Moreover, results reveal that miRNAs with high-influence on the disrupted protein complexes are diagnostic and prognostic biomarker candidates for prostate cancer progression. The observation of miRNA-mediated protein complex regulation and miRNA-mediated pathway regulation, with partial experimental verification from previous studies, demonstrates that our framework is a promising approach for the identification of novel miRNAs and protein complexes related to disease progression.

## Introduction

Prostate cancer (PCa) is the most frequent male malignancy and the second cancer-related cause of death in Western countries [1]. Recently, considerable evidence has shown that non-coding RNAs in general [1] specifically miRNAs are implicated in PCa and are associated with its progression [2]–[6]. In particular, circulating miRNAs are promising biomarkers of PCa progression [7], [8]. Though there are only around 1000 miRNAs [9] in human, each only 18–22 bp in length, more than one hundred of them play a role in cancer [10], and they act as both oncogenes and tumor suppressors [11]. Thus, characterizing the role of miRNAs in PCa is crucial to understanding their function and possible utility for therapeutic purposes.

Recently, the cross-talk between miRNA-target networks and protein networks has been analyzed in several aspects [12]–[15]. For example, direct miRNA targets and their partners in protein-protein interactions (PPI) networks show significant modularity [14]. miRNAs have specific effects on the formation of protein complexes by selecting specific components of the complex [12], and some protein complexes are enriched with targets of specific miRNAs [13]. A positive correlation between protein connectivity and number of different targeting miRNAs was observed [15] indicating that hub proteins require more miRNA-mediated regulation. In addition, miRNAs can simultaneously regulate several proteins in the same functional module such as biological pathways. Furthermore, PPI network topological features are useful in filtering out false positive miRNA targets [16], and in prioritizing miRNAs implicated in prostate cancer [17]. This process is important to rank the significant miRNAs with a potential role in prostate cancer. Taken together, there is clear evidence of coordinated post-transcriptional regulation of protein complexes and pathways by miRNAs. However, the regulatory influence of miRNAs on genes whose encoded proteins are part of protein complexes or protein pathways that are implicated in cancer has not been thoroughly investigated.

To date, a number of mathematical models have been developed to infer miRNA-mRNA modules or modular networks using gene expression and miRNA-gene networks [18], [19]. For instance, SVD is a useful mathematical framework that has been applied in identifying implicated miRNA-mRNA modules in prostate cancer [20], in addition to several areas of computational biology [21]–[23]. SVD is helpful for biologists to analyze and model genome-wide expression data, and reduce data dimensionality [22]. Given an 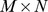 matrix 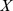, the singular value decomposition (SVD) of 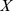 is its representation as 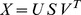, where 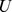 is an orthogonal 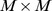 matrix, 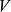 is an orthogonal 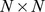 matrix, and for the diagonal matrix 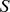, elements are non-negative numbers in descending order. The power of SVD resides in the three matrixes generated as a result of the decomposition. The squares of the singular values represent the relative importance of the entropy in 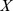 matrix. Utilizing this fact, SVD is used to rank genes based on the entropy they contribute to the gene expression data [23].

In the post-genomics era, a crucial task in molecular biology is to understand gene regulation in the context of biological networks. Since miRNA target proteins, among others, that are part of protein complexes and signaling pathways, it is important to study the miRNA-mediated regulation of protein complexes in disease progression. Using the protein network context of the miRNA targets adds another layer of information to consider for miRNA function characterization as miRNA influence on targets propagates through the protein network to affect multiple components of the pathway. Several studies reported regulation of functionally related proteins by miRNAs [12]–[15], but little is known about how miRNAs coordinately regulate protein complexes and pathways in cancer.

In this study we propose SVD-based computational framework to identify miRNA-protein complex modules that are dysregulated in cancer. miRNA-protein complex and miRNA-pathway modules refer to the proteins in the protein complex or pathway and the miRNAs targeting the genes encode them. Every module is represented as a matrix where rows are protein members and columns are targeting miRNAs. Every cell in the matrix represents the correlation between the expression profile of the miRNA and the expression profile of the protein. We anticipate that modules that have significant entropy change in their singular values between normal and cancer samples are functionally dysregulated. We applied the proposed computational framework to characterize experimentally verified protein complexes from the CORUM database [24], as well as from curated biological pathways from Molecular Signatures Database (MSigDB), and miRNA-target interactions to identify miRNA-mediated protein complexes and pathways dyregulation.

## Materials and Methods

### miRNA-targets interactions and protein complexes

Experimentally verified miRNA-target interactions were retrieved from two sources: MiRecords [25] and miRtarbase [26]. For protein complexes, we retrieved 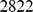 complexes from CORUM(last accessed May,2012), which provides a resource of manually annotated protein complexes from mammalian organisms [24]. Complexes of size less than 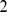 or complexes not targeted by any miRNA were removed as they do not form miRNA-protein complex modules. 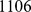 complexes remained in the study when using the miRNA-target interaction set. For biological pathways, we used curated pathways from Molecular Signatures Database (MSigDB) gene sets [27] that contain 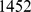 canonical pathway gene sets(last accessed August,2012).

### miRNA and target expression profiles in prostate cancer

mRNA and miRNA expression data was retrieved from the MSKCC Prostate Oncogenome Project, available at the Gene Expression Omnibus (GEO accession number: GSE21032). This data contains mRNA and miRNA expression levels of 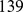 matched samples. This data that we will refer to as the Taylor data is used to build the miRNA-protein complex modules. We also used localized prostate cancer miRNA expression data from two independent experiments (GSE23022 [28], NCI-60 [29]) to validate the diagnostic significance of the miRNAs found to influence protein complexes. The first dataset contains 20 normal and 20 tumor samples, and the latter contains 6 normal and 6 tumor samples. Three independent prostate mRNA expression datasets from Arul *et al.* [30], Yu *et al.* [31] and the Swedish prostate cohort [32] are used. The Arul *et al.* data contains 6 normal, 7 primary, and 6 metastasis samples; the Yu *et al.* prostate data contains 17 normal, 63 primary, and 24 metastasis; and the Swedish prostate data contains 281 prostate cancer samples with 116 lethal and 165 indolent samples. The Swedish cohort data was used to validate the prognostic value of affected protein complexes. The Yu *et al.* and the Arul *et al.* data sets are used to validate the diagnostic significance of the influenced protein complexes. Non-prostate cancer miRNA expression data from NCI-60 [29] and breast cancer mRNA expression data from Swedish breast cohort [33], containing 159 tumor samples with clinical data, were also used to assess if the influenced miRNA-protein modules are prostate specific or they are dysregulated in other cancers as well.

### Defining miRNA-protein complex modules' entropy

For each miRNA-protein complex module, we construct a matrix 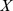 where rows (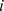) represent proteins in the complex or the pathway and columns (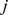) represent miRNAs that target at least one member of the complex. 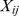 is defined as the mutual information [34] between the expression profile of protein 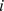 and the expression profile of miRNA 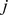 and is calculated as:
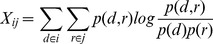
(1)

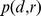
 is the joint probability density function (pdf) of 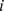 and 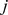, and 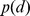 and 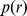 are the marginal pdfs of 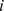 and 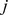 respectively. The probability distribution functions were estimated using kernel density estimates [35] as it showed to be superior to the histogram in terms of a better mean square error rate of convergence of the estimate.

**Figure 1:**
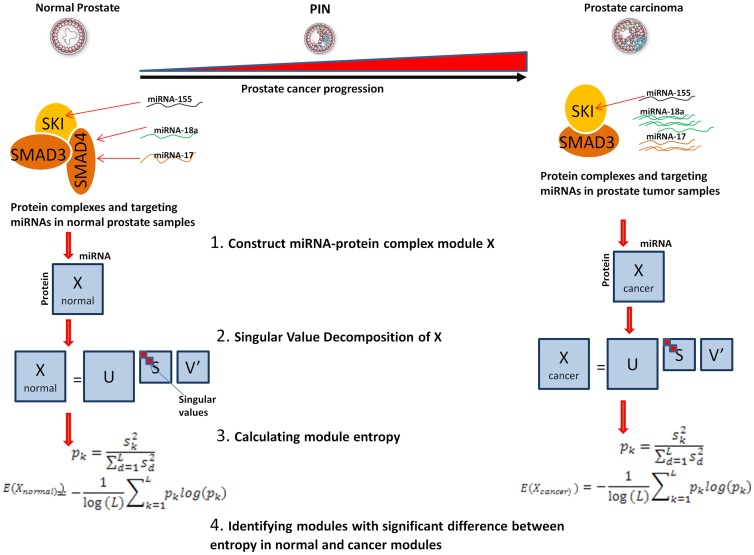
Overview of the proposed framework to construct miRNA-protein complex modules and calculate module entropy between cancer and normal states. Protein complexes and miRNAs are integrated to construct modules (X) from gene and miRNA expression data. The modules represent the mutual information between the expression of miRNAs and protein in the module. SVD is applied to decompose modules' matrix and Shannon entropy is calculated for each module in normal and cancer. The last step is to find modules with significant difference between normal and cancer entropy.

X is the mutual information matrix between all miRNAs and all genes in the complex module. Since we believe that when a miRNA target a gene in the complex (based on miRNA-target interaction), it may have indirect effect on the other members of the complex. Thus the matrix X does not distinguish between a miRNA that target a gene in the complex or not. The matrix X is based on the notion that if a miRNA target a gene in the complex, it has influence on the whole complex. The influence of miRNAs on each protein complex or pathway is calculated by decomposing 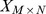 using Singular Value Decomposition (SVD) [23] into matrices 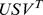 and compute the entropy of the matrix by summing the squares of the singular values in the 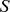 matrix. 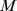 is the number of proteins in the protein complex, and 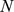 is the number of targeting miRNAs. The normalized relative significance of 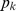 of the 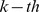 singular value in 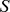 is calculated as
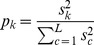
(2)and the Shannon entropy of the data, represented by 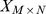, is calculated as:



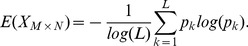
(3)Where 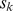 is the 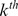 singular value, L is 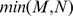. Here we anticipate that miRNA-protein complex modules that have significance difference in the entropy of the singular values of 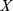 between normal and cancer samples are functionally dysregulated. The entropy of the singular values represents the dysregulation of the miRNA-protein complex modules. Figure 1 provides a brief description of the proposed framework. The first step is to construct the miRNA- protein modules by calculating the MI between all the expression of the proteins in the complex and the expression of the miRNA targeting them. For each stage of cancer (normal vs primary prostate cancer) we define the miRNA-protein modules. Second, we find the singular values of each matrix and calculate the entropy as the normalized sum of the squares of the singular values. Finally, we find the modules with significant difference between the modules representing the normal stage and the cancer stage.

### Identifying miRNA-coordinated protein complexes and pathways in prostate cancer progression

Using gene expression data for normal and cancer samples, we found 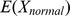 and 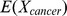 respectively for each module. We used the difference between the two values, 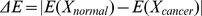, to assess module's influence by miRNAs. To assess the significance of the influence value, we randomly permuted protein complexes and pathways with the same size as the complex of interest 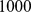 times, and found 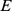 for both normal and cancer samples. 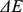 was calculated for the random permutations, and a p-value was computed for each complex and pathways with the observed 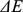 against the distribution of the 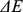 values generated from the random permutations. The 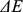 value represents the miRNAs' influence on protein complexes or pathways in prostate cancer progression; the higher the 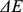, the more influenced the protein complex is. P-values were corrected using Bonferroni correction. Modules that are significantly dysregulated by miRNAs in prostate cancer progression were further characterized both functionally and clinically.

### Identifying downstream miRNA-mRNA interactions influences by dysregulated protein complexes

We next asked if there are downstream miRNA-target interactions influenced by the affected protein complexes. We defined downstream genes as those that are dependent (correlation conditional on protein complex dysregulation). To identify such conditional interactions, we used conditional mutual information between miRNAs and their experimentally validated targets from 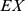 given the expression of the influenced protein components of the complex.

Given protein complex 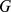 and its components, 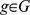. We calculated the conditional mutual information between each miRNA (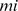) and target (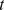) pair given the expression of protein 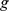, 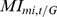, as described in [36]:
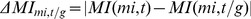
(4)

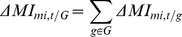
(5)


We then found p-value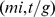 for each interaction (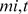) given a protein 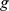 by permutating the expression profile of protein 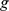 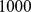 times. To find the p-value for each complex, p-value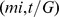, we converted the individual p-values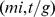, 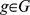, to 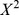 test statistics using Fishers method 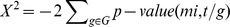.

## Results

Protein complexes and biological pathways that are influenced in prostate cancer as a result of coordinate miRNA regulation are identified and further functionally characterized.

### miRNA-influenced protein complexes modules

We first analyzed the influence of miRNAs on protein complexes in prostate cancer progression. We constructed miRNA protein modules by integrating expression data, miRNA-target interactions, and protein complexes (CORUM) as described in the methods section, and then identified the entropy change of each module in different prostate status(normal vs. cancer). Table 1 shows the full list of the most significant protein complexes influenced by miRNA regulation in prostate cancer (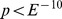) using the experimentally determined (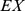) miRNA-target interactions. Bonferroni correction is used for multiple-testing correction. In total, 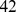 complexes are predicted to be influenced by miRNAs. Results reveal that complexes containing SMAD4 are significantly affected in prostate cancer progression, and that the SMAD6-HOXC8 complex is the most significantly influenced complex. This complex plays a role in transcriptional repression by inhibiting interactions between SMAD1 and HOXC8. The next two important complexes contain SMAD4, SKI, and SMAD3. Another set of miRNA-influenced complexes contain SIN3A, HDAC, and ARID4B; these complexes act as transcriptional repressors on MYC responsive genes and antagonize MYC oncogenic activity, and they play a role in histone deacytelation, which is important in gene expression control. Several other complexes containing RBL1 and ARID4B, which has a sequence similar to RBL1, are significantly affected. Most of the complexes predicted to be influenced by miRNAs are of size less than 5. Two complexes; namely, LINC complex (Corum ID: 5589) and SAP complex (Corum ID: 591) are predicted to be influenced by miRNAs. Interestingly, only RBL1 gene in LINC complex and ARID4B in SAP complex are directly targeted by miRNAs, suggesting that disruption of one protein by multiple miRNAs could lead to disruption of the protein complex. SAP complex is composed of histone binding and histone deacetylation proteins suggesting a key role of epigenetic changes in prostate progression. A list of the most significant protein complexes and their targeting miRNAs is shown in Table S1. Visualizing the heatmap of the complex modules (protein and miRNAs) reveal that they can together define expression pattern for Primary cancer and metastatic cancer (Figure S1 and S2 in File S1).

**Table 1:** Significant miRNA-coordinated protein complexes using experimentally validated miRNA-target interactions set 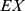.

CORUM ID	CORUM Complex Name	p value	Complex size	# miRNAs	Biological Process
*4089*	*SMAD6-HOXC8 complex*	*1.01E-199*	*2*	*4*	*DNA binding*
*3959*	*SMAD3-SMAD4-cSKI*	*3.94E-104*	*3*	*9*	*TGF-beta-receptor signalling pathway*
*3740*	*SKI-SMAD3-SMAD4 pentameric complex*	*6.19E-92*	*3*	*9*	*TGF-beta-receptor signalling pathway*
*2447*	*ITGA9-ITGB1-ADAM12 complex*	*6.55E-88*	*3*	*4*	*Cell-cell adhesion*
*3200*	*SMAD4-SKI complex*	*2.40E-75*	*2*	*9*	*TGF-beta-receptor signalling pathway*
*5589*	*LINC complex*	*3.74E-67*	*7*	*3*	*G2/M transition of mitotic cell cycle*
*531*	*XPA-ERCC1-ERCC4 complex*	*3.29E-66*	*2*	*3*	*DNA repair*
*2590*	*FOXO1-FHL2-SIRT1 complex*	*2.15E-59*	*3*	*11*	*modification by acetylation, deacetylation*
*2763*	*MBD1-Suv39h1-HP1 complex*	*1.61E-47*	*3*	*2*	*posttranslational modification of amino acids*
*3206*	*SMAD4-SKI-NCOR complex*	*7.15E-44*	*3*	*9*	*TGF-beta-receptor signalling pathway*
*5382*	*ARNT-HIF1A complex*	*3.53E-38*	*3*	*5*	*Transcription activation*
*5409*	*TIAM1-GRIN1 complex*	*2.28E-36*	*2*	*2*	*Cell growth*
*5158*	*SMARCA2/BRM-BAF57-MECP2 complex*	*2.03E-35*	*3*	*5*	*Transcription repression*
*5281*	*Cell-cell junction complex (CDH1-CTNNB1)*	*6.93E-35*	*2*	*2*	*cadherin mediated signalling pathway*
*3739*	*SKI-SMAD2-SMAD4 pentameric complex*	*1.13E-29*	*3*	*10*	*TGF-beta-receptor signalling pathway*
*5190*	*TIAM1-EFNB1-EPHA2 complex*	*1.19E-27*	*3*	*4*	*Neurogenesis*
*591*	*SAP complex (Sin3-associated protein complex)*	*1.74E-26*	*8*	*4*	*Modification by acetylation, deacetylation*
*3197*	*SMAD4-SNO-SKI complex*	*3.57E-26*	*3*	*9*	*TGF-beta-receptor signalling pathway*
*2761*	*SMAD3-SMAD4-FOXO1 complex*	*1.82E-24*	*3*	*13*	*TGF-beta-receptor signalling pathway*
*903*	*RET-Rai complex*	*1.53E-22*	*5*	*2*	*Transmembrane signal transduction*
*1096*	*SNX complex*	*1.75E-22*	*4*	*2*	*Receptor enzyme mediated signalling*
*695*	*SIN3-HDAC-SAP30-ARID4 complex*	*1.80E-22*	*4*	*80*	*Modification by acetylation, deacetylation*
*592*	*SAP complex (Sin3-associated protein complex)*	*2.78E-19*	*9*	*4*	*Modification by acetylation, deacetylation*
*738*	*SIN3-ING1b complex I*	*4.30E-19*	*9*	*4*	*Modification by acetylation, deacetylation*
*2880*	*SCF subcomplex*	*4.44E-19*	*3*	*7*	*Modification by phosphorylation,ubiquitination*
*862*	*DNMT1-G9a complex*	*4.29E-18*	*3*	*6*	*DNA methylation and posttranslational modification of amino acids*
*5920*	*KSR1-RAF1-MEK complex*	*5.63E-18*	*4*	*4*	*MAPKKK cascade*
*691*	*SIN3-SAP25 complex*	*2.28E-17*	*11*	*9*	*Modification by acetylation*
*1539*	*G protein complex*	*1.99E-15*	*3*	*2*	*G-protein coupled receptor signalling pathway*
*3046*	*hs4 enhancer complex (slow migrating complex)*	*2.43E-15*	*2*	*2*	*NIK-I-kappaB/NF-kappaB cascade*
*1490*	*DAXX-DNMT1-DMAP1 complex*	*4.98E-15*	*3*	*6*	*Transcription repression*
*521*	*Polycystin-1-E-cadherin-beta-catenin complex*	*1.45E-14*	*3*	*2*	*Intercellular junction*
*2593*	*FOXO3-SIRT1 complex*	*1.04E-12*	*2*	*8*	*Anti-apoptosis*
*5184*	*SWI/SNF chromatin-remodeling complex*	*1.20E-12*	*5*	*5*	*Transcription repression*
*836*	*20S methyltransferase core complex*	*1.68E-12*	*2*	*5*	*Posttranslational modification of amino acids*
*3086*	*CCND3-CDK4 complex*	*2.51E-12*	*2*	*8*	*Modification by phosphorylation*
*1473*	*E2F5-RB2-DP1 complex*	*1.47E-11*	*3*	*7*	*Transription*
*5171*	*SH3KBP1-CBLB-EGFR complex*	*5.13E-11*	*3*	*6*	*Transmembrane receptor protein tyrosine kinase signalling pathways*
*2944*	*Notch1-p56lck-PI3K complex*	*6.67E-11*	*3*	*13*	*Notch-receptor signalling pathway*

The table showed the corrected p-value, the size of the complex and the number of miRNAs targeting the complex.

### Functional analysis of miRNA-influenced protein complexes

**Figure 2:**
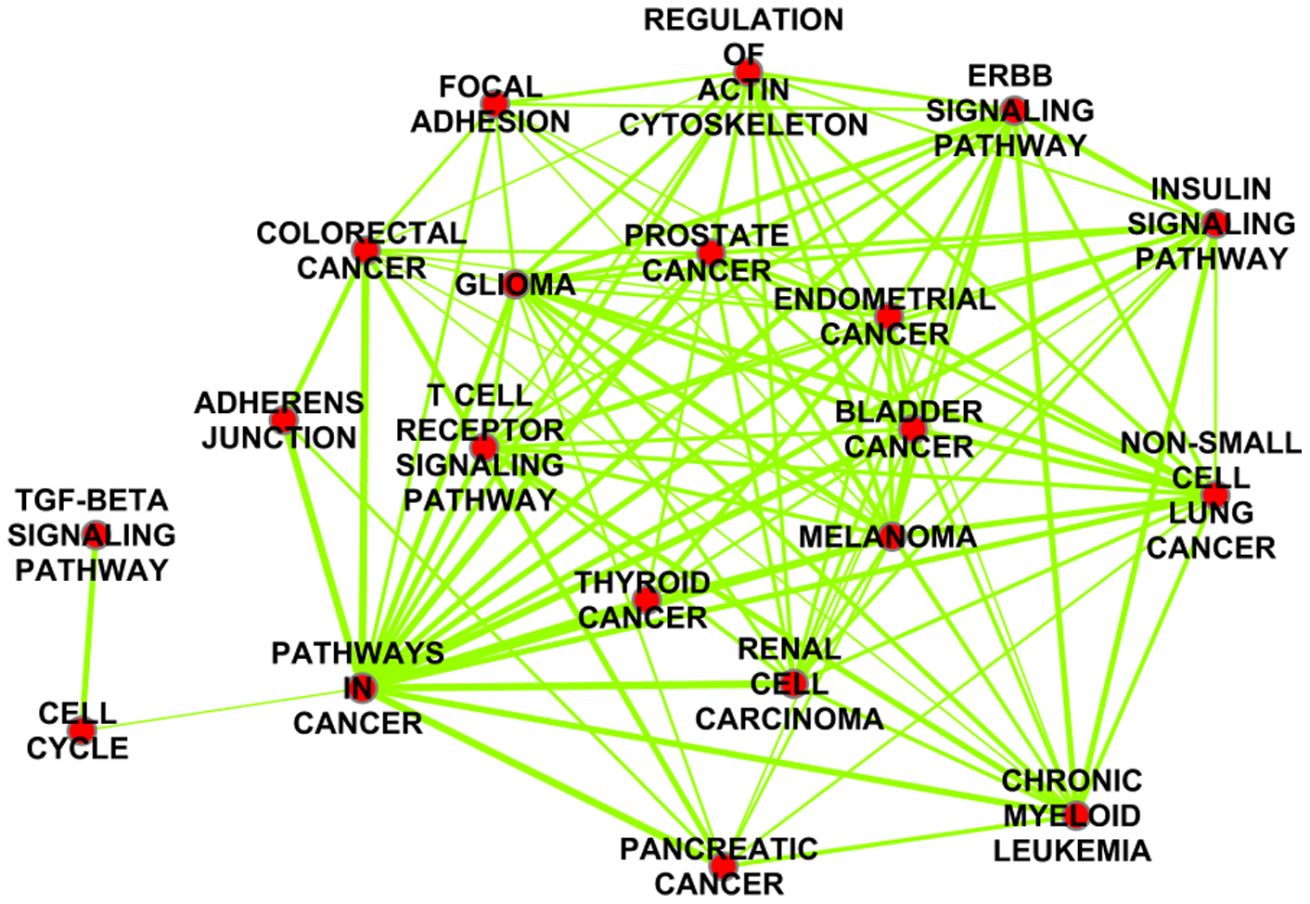
Pathway Enrichment Map of dyregulated protein complexes. Pathway Enrichment Map of dyregulated protein complexes. We extracted the protein members of the dysregulated protein complexes and found enriched pathways using DAVID online tool.To visualize enrichment map of pathways, we used Enrichment Map Cytoscape plugin [47] to visualize enriched pathways. Nodes in this figure represents enriched pathways, links between nodes represent the fraction of overlap between them. The darker the node the more enriched the pathway is, and the thicker the link, the more significant the overlap is.

We performed functional analysis on the miRNA-influenced proteins complexes by analyzing the biological processes they are involved in. We performed functional analysis on the components of the 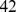 complexes in Table 1 using the DAVID online tool [37] available at (http://david.abcc.ncifcrf.gov/) ( 84 proteins were functionally characterized). Benjamini multiple-testing correction was applied for significant enrichment analysis. Functional analysis demonstrated that the components of the complexes are enriched with three major biological terms, phosphorylation (p = 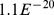), transcriptional regulation (p = 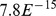) and acetylation (p = 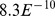). Proteins in the complexes are enriched in Dwarfin (p = 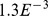), MAD homolog (p = 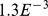), SMAD (p = 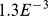) and tyrosine protein kinase (p = 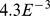) domains. The proteins are enriched in the TGF-B signaling pathway (p = 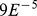), pathways in cancer (p = 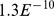), prostate cancer (p = 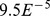), and other specific cancers (Figure 2). Analyzing the molecular function of the proteins supported that the influenced complexes' components are involved in transcription regulation ( p = 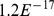), SMAD binding (p = 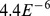), protein kinase activity (p = 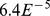) and DNA binding (p = 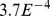). We then analyzed the pathways of the miRNA targets in the complexes in the background of all the miRNA validated targets. We used DAVID to find the enriched terms in the miRNA targets in the 82 protein in the background of the miRNA validated targets. We found the complexes enriched in pathways in cancer (p = 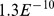), prostate cancer (p = 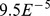) and bladder cancer (p = 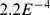).

### Characterizing the relationship between the complex size and the complex entropy

**Figure 3:**
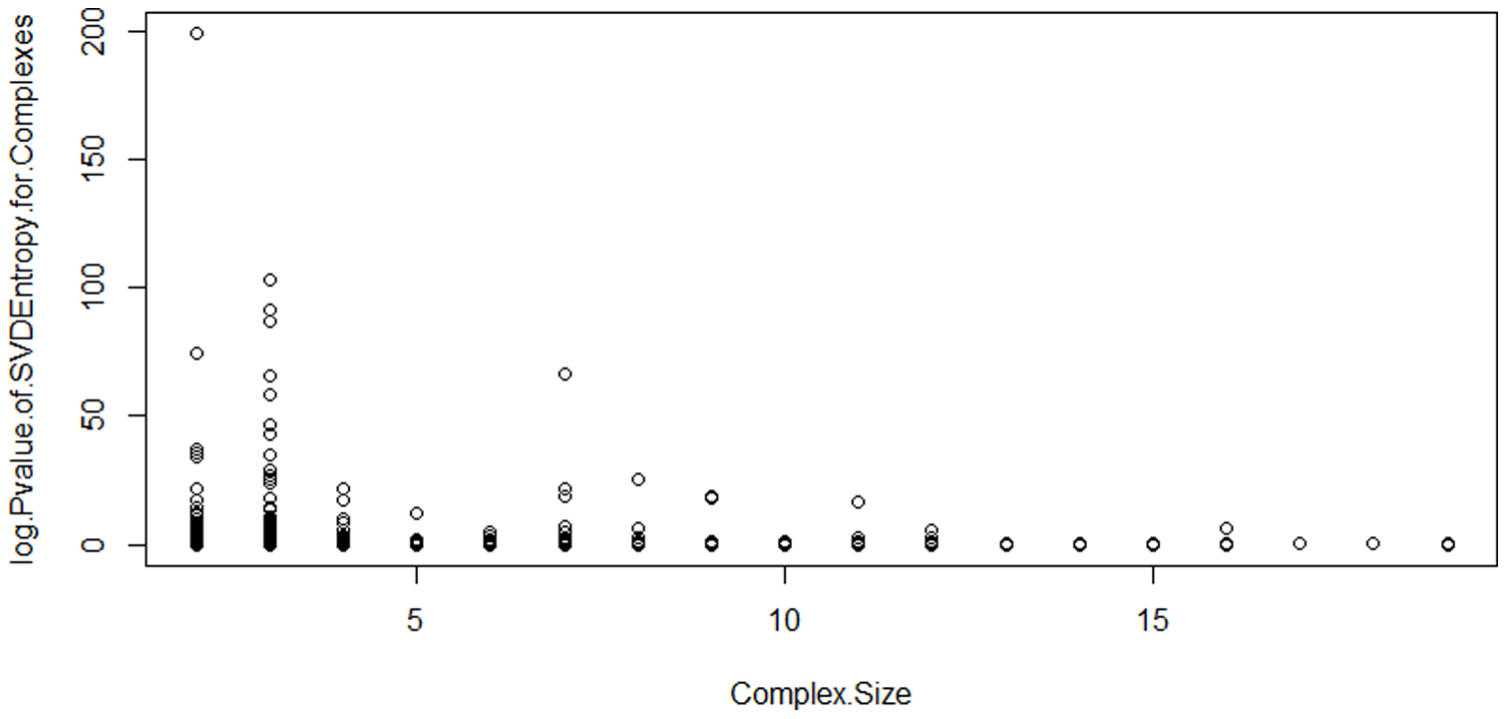
Correlation between protein complex size and the significance of the SVD based entropy. Using the experimental miRNA-target interaction to assess the significance of miRNA-mediated dysregulation of protein complexes, we analyzed the relationship between the complex size and the p.value generated by our framework.We found that complexes f size 2, 3 and 7 have the most significant pvalue, and complexes of size less than 10 are not very significant.

The entropy p.values of the protein complexes varied between 0.8 to 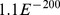. One of the questions we asked is whether the entropy values are driven by the complex size. We found that complexes of size 2, 3 and 7 have the most significant pvalue, and complexes of size greater than 10 are not very significant (Figure 3). There are different biological interpretations for this observation. One is that smaller complexes are more easily targeted by miRNAs; however, when one protein of a larger complex, the complex may still be functional but with less efficiency. Another interesting observation is that there is no correlation between the size of the protein complex and the number of miRNAs targeting the protein in the complex (Table 1).

### miRNA-influenced canonical pathway modules

To find the influence of miRNAs on pathways, we demonstrated the applicability of the framework on curated protein pathways from the MSigDB gene set database. We asked how the the size of the protein modules may affect the entropy value of the miRNA influence. We used miRNA-target interactions to find the miRNA influence on pathways. Table 2 shows the pathways from MSigDB that are significantly influenced by miRNAs in PCa; results reveal that Syndecan-mediated and RAS signaling pathways are highly influenced by miRNAs. The NF-kB mediated pathway involving adaptor proteins MYD88 and TRAF6, that are involved in the Toll-like receptor and IL-1 receptor signaling pathways, is also influenced by miRNAs. Chromatin maintenance and RNA-polymerase mediated transcription are also influenced by miRNAs in prostate cancer. From the miRNA-target interactions, we found 54 miRNAs that target the significant pathways; 24 of them target more than one member of the pathway. miRNA-1, miRNA-7b, and miRNA-16 were found to target more than 5 different members of the same pathway, suggesting that these three miRNAs are key regulators of prostate cancer.

**Table 2:** Table 2. Canonical pathways influenced by miRNAs in prostate cancer.

Canonical pathway	Corrected p-value
*REACTOME-ABCA-TRANSPORTERS-IN-LIPID-HOMEOSTASIS*	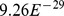
*REACTOME-MYD88-MAL-CASCADE-INITIATED-ON-PLASMA-MEMBRANE*	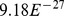
*REACTOME-ACETYLCHOLINE-NEUROTRANSMITTER-RELEASE-CYCLE*	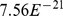
*PID-RAS-PATHWAY*	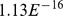
*PID-SYNDECAN-2-PATHWAY*	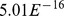
*PID-LYMPHANGIOGENESIS-PATHWAY*	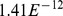
*REACTOME-TRAF6-MEDIATED-INDUCTION-OF-NFKB-AND-MAP-KINASES-UPON-TLR7-8-OR-9-ACTIVATION*	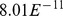
*REACTOME-ACTIVATED-AMPK-STIMULATES-FATTY-ACID-OXIDATION-IN-MUSCLE*	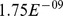
*MIPS-CRSP-MEDIATOR-2-COMPLEX*	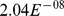
*BIOCARTA-EDG1-PATHWAY*	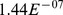
*PID-SYNDECAN-1-PATHWAY*	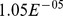
*KEGG-GLUTATHIONE-METABOLISM*	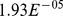
*REACTOME-CHONDROITIN-SULFATE-DERMATAN-SULFATE-METABOLISM*	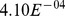
*REACTOME-CYCLIN-E-ASSOCIATED-EVENTS-DURING-G1-S-TRANSITION*	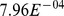
*REACTOME-SEMA3A-PLEXIN-REPULSION-SIGNALING-BY-INHIBITING-INTEGRIN-ADHESION*	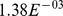
*BIOCARTA-BARR-MAPK-PATHWAY*	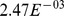
*REACTOME-CHROMOSOME-MAINTENANCE*	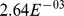
*REACTOME-RNA-POL-I-TRANSCRIPTION-INITIATION*	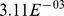
*PID-VEGFR1-2-PATHWAY*	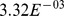
*PID-PI3KPLCTRKPATHWAY*	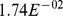

### miRNAs influencing protein complexes have a role in prostate cancer progression

We then investigated the functional role of 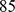 miRNAs that target the 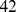 protein complexes. Only 66 miRNAs were present in the Taylor gene expression data. We generated a list of 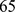 miRNAs from a thorough literature search for miRNAs involved in prostate cancer. 45% of the 66 miRNAs are in common with the 65 miRNA (p = 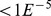) that have an experimentally validated functional role in prostate cancer progression, such as miR-1, miR-106b, miR-221, miR-222, miR-96, and miR-182. (see Table S2).

### Prognostic value of miRNA-protein complexes modules

**Figure 4:**
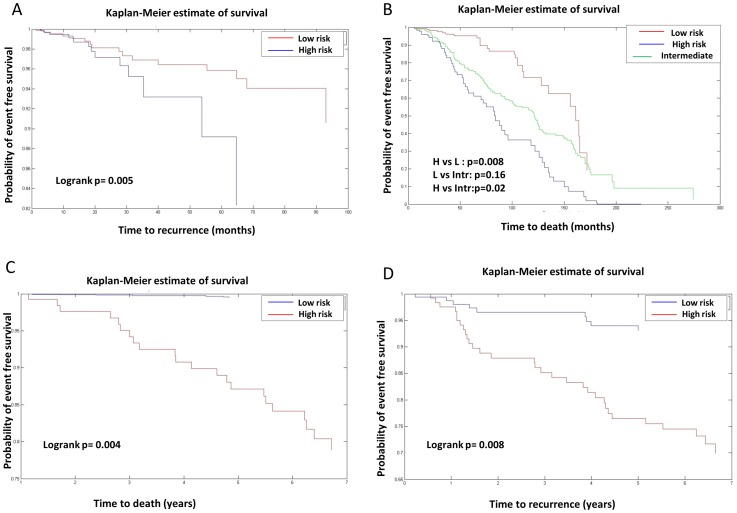
Kaplan-Meier plots for the 23 proteins from the Taylor and Swedish data. A. Samples were grouped into two groups based on the expression of the 23 proteins from the Taylor mRNA data and then logrank test was applied to assess separation significance (p = 0.005). B. Samples from the Swedish prostate cohort were grouped into three groups using the expression of the 23 proteins. The resulted three groups are significantly separated which shows the prognostic power of the 23 proteins (low risk vs. high risk (p = 0.008), low risk vs. intermediate risk (p = 0.16), high risk vs. intermediate risk ( p = 0.02)). C. Samples from the Swedish breast cohort were grouped into two groups based on the expression of the 23 proteins. The two groups have distinct death specific association (p = 0.004). D.Samples from the Swedish breast cohort were grouped into two groups based on the expression of the 23 proteins. The two groups have distinct cancer recurrence profile (p = 0.008).

In this section we characterize the prognostic value (cancer recurrence and time to death) of the miRNA-protein complex modules. We first retrieved the expression of the 84 proteins, that are part of the 42 complexes in Table 1, from both the Taylor and the Swedish prostate data. We also extracted the miRNA expression of the 85 miRNAs that target the 84 proteins from the Taylor prostate data. Initially we clustered the protein and miRNA samples into two groups using k-means clustering, and then use logrank and COX-hazard regression test to assess the clinical significance of the separation. The objective here is to show that the protein complex members can stratify patients into clinically distinct groups. Unfortunately, results were not significant; clustering the patients based on the 85 miRNAs into two sets gave 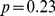 from the Taylor miRNA data. On the other hand, clustering the 84 proteins into two groups based on the Taylor mRNA data gave 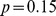, and 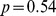 based on the Swedish data. To extract more accurate prognostic biomarkers from these lists, we performed univariate COX-hazard regression analysis and then selected proteins with significant p-value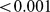. The set of 84 proteins was reduced to 23 proteins and miRNA set was reduced to 21 miRNAs (Table 3). We then performed clustering based on the expression of proteins and miRNAs in the reduced set and characterized their clinical significance. For the 23 proteins, the clustered set of patients in the Taylor data are significantly separated (p = 0.005) (Figure 4A). As a negative control, we randomly selected 23 proteins 1000 times and repeated the clustering and logrank test, achieving an average of p = 0.26. Further grouping the samples into three sets demonstrated more significant separation between high-risk and low-risk patients (p = 0.00088) (Figure S3 in File S1). To further test the prognostic value of the 23 genes on the Swedish dataset (independent data that was not used to identify miRNA-influenced protein complexes), we used their expression values from the Swedish data, and grouped samples into two groups that were not significantly separated (p = 0.5). However, when we clustered samples in the Swedish across the 23 proteins into three groups, we found significant separation into low-risk, intermediate risk and high risk patients (Figure 4B). High-risk patients are significantly separated from low-risk patients (p = 0.008) compared to the average of 1000 random permutations of the samples (p = 0.63).

**Table 3:** Prognostic proteins and prognostic miRNAs that were extracted from the 84 and 85 protein and miRNA lists respectively based on univariate regression analysis.

Prognostic proteins	Prognostic miRNAs
*ADAM12*	*hsa-miR-1*
*ARID4B*	*hsa-miR-106b*
*E2F5*	*hsa-miR-125b*
*EGFR*	*hsa-miR-145*
*FHL2*	*hsa-miR-155*
*HOXC8*	*hsa-miR-16*
*MYBL2*	*hsa-miR-182*
*PIK3R1*	*hsa-miR-18a*
*RBBP7*	*hsa-miR-194*
*SAP18*	*hsa-miR-195*
*SIN3A*	*hsa-miR-199b-3p*
*SIRT1*	*hsa-miR-204*
*SNX1*	*hsa-miR-20b*
*SMAD3*	*hsa-miR-210*
*SMAD4*	*hsa-miR-221*
*DNMT1*	*hsa-miR-23b*
*HDAC1*	*hsa-miR-26b*
*HDAC2*	*hsa-miR-27b*
*ITGB1*	*hsa-miR-29b*
*MYBL2*	*hsa-miR-31*
*RBBP4*	*hsa-miR-92a*
*SMAD6*	
*TFDP1*	

When we functionally analyzed the enriched terms in the 23 proteins, we found that they are enriched in several cancer pathways such as Cell cycle (p = 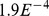), TGF-beta pathway (p = 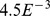), Chronic myeloid Leukemia (p = 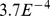),and Notch signaling (p = 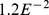). In addition, the genes were enriched in transcription regulation biological process (p = 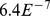).

To test the prognostic value of the 23 proteins for other cancer types, we used breast data from the Swedish breast cohort. Grouping the samples into two sets using the 23 proteins reveals significant association with cancer-specific death and cancer recurrence (Figures 4C-D). We also used GOBO online tool [38] (http://co.bmc.lu.se/gobo) to associate the expression of the proteins with distant metastasis free survival across more than 1200 samples with different genotypes. The 23 proteins are found to be associated with breast cancer metastasis across all samples (p = 0.0076) (Figure S4A in File S1). Results also reveal that the 23 proteins are more closely associated with metastasis in the ER-positive (p = 0.00057) (Figure S4B in File S1) and the LN-negative breast cancer (p = 0.004) (Figure S4C in File S1) subtypes.

To characterize the prognostic value of the 21 influencing miRNAs, we extracted their expression data from the Taylor miRNA data and clustered the samples into two groups. The 21 miRNAs harbour significant prognostic value as they lead to significant separation between the two resulted patient sets (p = 0.00004, 1000 random sets gave p = 0.11) (Figure S5 in File S1). When samples were grouped into three groups across the 21 miRNAs, very significant separation between the low-risk and high-risk samples (p = 0.00021, 1000 random sets gave p = 0.28) (Figure S6 in File S1) is found. The prognostic power of the 21 miRNAs was compared to 94 miRAs differentially expressed between tumor and normal in the Taylor data, and 50 miRNAs differentially expressed between aggressive prostate cancer and non-aggressive cancer. The 94 miRNAs have a logrank p = 0.019 and the 50 miRNAs have logrank p = 0.00046. This result suggests that miRNAs that influence protein complexes are significant prognostic biomarkers.

In summary, results reveal that miRNAs that coordinately regulate protein complexes are valuable prognostic biomarkers. In addition, protein complexes dysregulated by miRNAs are prognostic biomarkers that are candidates as therapeutic targets for prostate cancer treatment.

### Validating the diagnostic power of the influenced protein complexes and miRNAs on independent expression data

To characterize the diagnostic role of the miRNA-influenced protein complexes and targeting miRNAs, we validated their ability to distinguish tumor samples from non-tumor samples using independent mRNA and miRNA expression data sets. A linear support vector machine with 10-fold cross validation was used to accurately predict the class label of patients (Normal, Primary or Metastasis). The SVM classifier takes expression data of patients across the miRNA-influenced proteins and aims to predict the class of the patients using the expression data. Here cross-validation is used to assess the performance of the model due to the lack of additional independent samples. Results (Table 4) reveal that the SVM, using the expression level of the proteins in the miRNA-influenced protein complexes, successfully separated primary from normal samples (85%) and metastasis from primary samples (100%) in the Arul *et al.* data. The proteins also classified primary and normal samples (80%) and metastasis vs. primary cancer (83%) in the Yu *et al.* data. Using the hierarchical clustering forthe Taylor data (Figure S7 in File S1), results show that mets samples are clearly separated from primary and normal samples.

**Table 4:** SVM classification with 10-fold cross validation to classify samples into normal, primary or metastasis using protein or miRNA expression profiles.

Protein complexes	miRNA
**Arul data**	**GSE23022**
*Primary vs. Normal: 85%*	*Primary vs. Normal:77.5%*
*Primary vs. Metastasis: 100%*	
*Normal vs. Metastasis: 100%*	
	*NCI60 (Normal vs. Primary tumor)*
**Yu data**	*Prostate: 91%*
*Primary vs. Normal: 80%*	*Colon:100%*
*Primary vs. Metastasis: 83%*	*Kidney: 100%*
*Normal vs. Metastasis: 76%*	*Lung: 100%*
	*Breast 100%*

To characterize the influential miRNAs and their role in prostate cancer progression, we validated the diagnostic power of the miRNAs on two independent prostate miRNA expression data sets (GSE23022, NCI-60). Classification results reveal that the influential miRNAs are robust diagnostic biomarkers as they are able to separate tumor from normal samples in GSE23022 (77.5%, random sets of miRNAs gave 54%) and NCI-60 data sets (91%, 63% using random sets) (Figure S8 in File S1). Additionally, the diagnostic power of the miRNAs was validated in non-prostate cancer (Colon(100%, 85% using random sets), Kidney(100%, 88% using random sets), Lung(100%, 86% using random set) and breast(100%, 90% using random set)). Interestingly, SVM, using the expression level of the miRNA set, was able to successfully separate tumor vs. normal samples across all the various cancer types (Table 4), indicating that these miRNAs might play a global role in regulating protein complexes in cancer.

**Figure 5:**
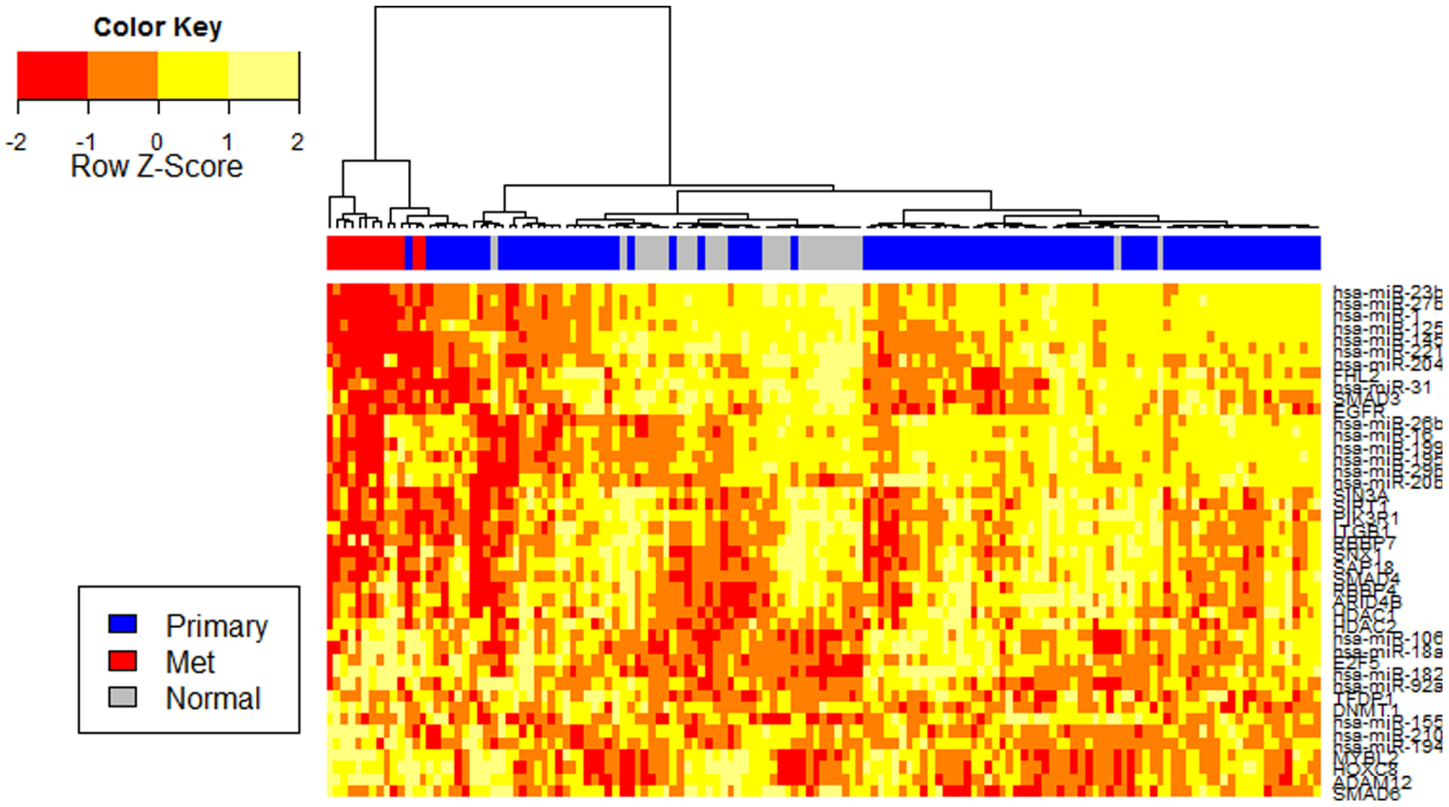
Heatmap of the 23 protein and 21 miRNA from Taylor data. We extracted the expression of 21 miRNAs and 23 proteins from Taylor data and then used hierarchical clustering to cluster samples. Results show that Mets samples are fully segregated from the normal and PCa samples. Normal samples tend to be grouped together and have a distinct expression profile.

We even got better classification for the Taylor data when we combined the 23 proteins and the 21 miRNAs. Figure 5 shows the heatmap of the combined proteins and miRNAs. The heatmap shows that the Mets samples are well segregated from the rest of the samples, and the normal samples are well clustered together. This result indicates that the modules are related to prostate cancer progression.

### Characterizing the downstream effect of dysregulated protein complexes

**Figure 6:**
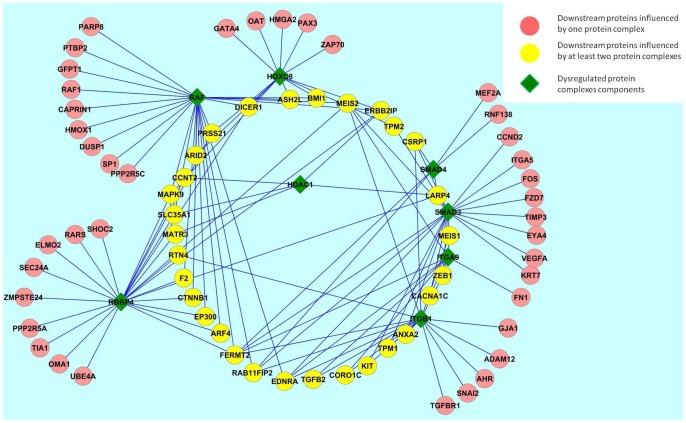
Network of dysregulated complexes and influenced proteins. Dysregulated protein complexes have a downstream effect on other proteins that affect their associations with miRNAs. We identified miRNA-target interactions influenced by the dysregulated protein complexes and then built a network between dysregulated complexes and proteins. Green diamonds represent the components of the dysregulated complexes. Circles represent proteins whose miRNA interactions are influenced by the dysregulated protein. An edge between a circle and a diamond proteins means that the dysregulation of diamond protein have a downstream effect on the ability of miRNAs to target circle proteins. Yellow circle proteins are proteins that are influenced by more than one protein complex.

We next asked if there are miRNA-target interactions that are affected by the dysregulated protein complexes. We selected the protein complexes that are highly dysregulated based on the results from Table 1: G-protein complex, SKI-SMAD3-SMAD4, ITGA9-ITGB1-ADAM12, SMAD6-HOXC8, SIN3-ING1b, and SIN3-HDAC-SAP30-ARID4 complexes. To characterize the downstream effect of the dysregulated protein complexes on miRNA-target interactions, we used conditional mutual information to assess the significance of the conditional dependence of the expression profiles of the miRNA-target interactions on the expression profiles of the complex protein members as described in Equation 4. Table S3 shows the miRNA-target interactions dependent on the protein complexes we are interested in. We found that miRNA-1 and miRNA-16 interactions are significantly dependent on the protein RAF, which is part of the G-protein complex. MEIS2-miRNA-204, FOS-miRNA221/222 and miRNA-1 are dependent on SMAD3 that is part of SKI-SMAD3-SMAD4 complex. Furthermore, MEIS1-miRNA-204, MEIS2-miRNA-204, ZEB1-miRNA-200c, and TPM1-miRNA-1 are dependent on ITGA9 and ITGB1. Lastly, we found that miRNA-200b/a are dependent on HDAC1 and miRNA-16 is dependent on RBBP4 that is member of SIN3-HDAC-SAP30-ARID4 complexes, suggesting that these miRNAs are key players of miRNA-mediated regulation in prostate cancer. Using these dependencies, we built a protein-protein network representing miRNA-coordinated regulation. Two proteins are connected if the interaction between one of them and a miRNA is conditionally dependent on the other protein (Figure 6). For a full list of interactions dependent on dysregulated protein complexes see Table S3.

## Discussion

MicroRNA-mediated regulation is a crucial layer of gene regulation that has influence on diverse biological processes. Using protein network context to characterize the mode of action of miRNAs is a new area of research in functional genomics that requires further investigation, to better understand miRNAs' influence on the target's protein context. Studying the miRNA-protein complex modules to characterize the diagnostic and prognostic role of miRNAs and protein interactions provide potential biomarkers and therapeutic targets relevant for prostate cancer diagnosis. A wide range of miRNAs and biological pathways are significantly altered in prostate cancer [39], but the influence of these miRNAs and their mode of action is not yet clear. In this work, we developed a computational framework that integrates expression data, miRNA-target network, and protein networks to identify miRNAs that coordinately regulate protein complexes in prostate cancer development, and characterize their role functionally and clinically. The developed framework explores the regulation of miRNAs and protein complexes or pathways. The framework also identifies miRNAs and proteins that are influenced by the dysregulated protein complexes. This framework can be easily applied in TF-mediated pathway dysregulation.

One of the factors that may affect identifying miRNA-protein interaction modules is the protein-interaction and miRNA-target data. Since both data are noisy and may lead to false discoveries, we used protein complexes data from CORUM database that are experimentally verified complexes. The second factor is the miRNA-target interaction data. Most of the computationally predicted interactions are noisy, thus we used experimentally validated miRNA-target interactions from miRecord and miRTarbase databases. Using experimentally validated interactions between proteins and miRNAs greatly helps to avoid noise in computational predictions.

Based on experimentally validated interactions, we identified 42 protein complexes that are dysregulated in prostate cancer progression as a result of a functional miRNA that targets at least one member of the protein complex. A mutual information measure was used to assess if correlation between the miRNA and its targets's expression profiles changes during cancer progression. Singular Value Decomposition has been widely applied to cluster gene expression data and assess the entropy gained in each module during cancer progression. Here, the entropy in the singular values of the modules represent the coordinated regulation of miRNAs on protein complexes. We found 42 complexes dysregulated in prostate cancer progression. The 42 complexes are composed of 84 unique genes that are not all prognostic. Using cox-regression the 84 set was reduced to 23 genes that are significantly prognostic to predict cancer recurrence. The 23 genes are distributed across most of the 42 complexes. We noticed some genes in the 23 set (like SMAD4, SIN3A, and others) are common among multiple complexes and thus this could explain why the number of reduced genes (23) is less than the number of complexes (42). We found two major types of complexes that are affected by miRNAs during cancer progression; the first contains SMAD3, SMAD2, SMAD4, SMAD6, FOXO1, HOXC8, and SKIL, which are connected to AKT1, which is in turn a key modulator of TGF-B signaling pathway [40]. Dysregulation of this pathway causes key changes in cell proliferation and growth. The second significant set of complexes contains SIN3A, RBBP7, HDAC1, HDAC2, SAP30, and SAP25 that participate in two important pathways. SIN3A is linked to MAX and MXD1, which are key regulators of MYC, acting as transcriptional repressors by interacting with MXI1 and then tethering SIN3A to DNA to repress transcription. The second important pathway is histone acytelation and deacetylation are highly dysregulated as many proteins RBBP7, RBBP4, HDAC1, HDAC9, HDAC2, SAP30, SAP25, and NCOR1 in these processes contribute to promote histone deacetylation and consequently transcriptional repression and nucleosome remodeling. Another set of proteins that play a key role in chromatin remodling is DMAP1 and DNMT1, which interact with HDAC2 to promote histone modification and transcriptional repression by methylating DNA and histones during cell replication. DNMT1 and HDAC1 levels are upregulated in prostate cancer, suggesting that they play roles in the inactivation of various critical genes via DNA methylation-induced chromatin remodeling [41]. Inhibitors of HDACs have emerged as potent anti-cancer agents; more than 100 clinical trials are ongoing with HDAC inhibitors as monotherapy or in various combination therapies [42]. This reveals that miRNA influence on epigenetic gene regulation is a key layer in gene expression control in prostate cancer.

We next asked if the identified dysregulated protein complexes are data specific, and extracted their expression from two independent data sets (Arul *et al.* and Yu *et al.*) and found that SVM correctly classify most of the samples using the expression of these proteins. This indicates that these proteins are dysregulated in prostate cancer. We further characterized their clinical significance and found that they are associated with cancer-specific death as they could separate high-risk from low-risk patients. We further tested the dysregulated protein on non-prostate data (Swedish breast cohort) and found that proteins are associated with cancer-specific death, tumor recurrence, and metastasis, indicating that these miRNA-influenced protein complexes could be dysregulated in multiple cancer types.

We further tested the diagnostic and prognostic ability of the regulating miRNAs and found that they are also and significantly accurate diagnostic biomarkers associated with prostate cancer recurrence. We used miRNA expression of other types of cancer (Lung, Kidney, Breast, Colon) and showed accurate diagnostic performance, concluding that miRNA-protein complex modules are diagnostic and prognostic biomarkers that could be used for therapeutic purposes.

We next sought to assess if the dysregulated protein complexes have a consequence effect on the expression of the downstream genes. Our results reveal that miRNA-1, miRNA-204, miRNA-16, and miRNA-200b are dysregulated due to the dysregulation of protein complexes identified. Finally we generated a network between dtsregulated protein complex members and proteins targeted by miRNAs based on conditional dependency. This helps to identify new proteins that are downstream of the miRNA-influenced protein complexes and are regulated through a miRNA but may not be part of known protein complexes, such as *MEIS1, MEIS2, ZEB1, TPM1, DICER2, TPM2*.

Another significant factor that should be considered is the size of the protein complexes used in this study. The protein complexes we used from CORUM are generally small (3-10 proteins) and may have an effect on the miRNA-mediated influence. Thus, considering larger functionally related proteins like signaling pathways proteins may support that our method is effective to find miRNA-influenced protein complexes. To investigate the influence of miRNAs on protein pathways, we constructed miRNA-protein pathways modules and found the most significantly miRNA-influenced pathways. The results show that RAS-mediated, NF-kB pathways and Syndecan-mediated pathways are highly influenced by miRNAs. Syndecan-1 is downregulated in prostate carcinoma, and transfection expression inhibits cancer growth [43]. Interestingly, there is no overlap between the pathways governing the dysregulated protein complexes and the dysregulated pathways identified. This may depend on several factors like number of miRNAs targeting each complex and the size of the complex itself. We found 54 miRNAs that target the pathways, three of which, miRNA-1, miRNA-7b, and miRNA-16 target more than 5 members of the same pathway. They were also found to target the physical protein complexes, suggesting a vital role of miRNA-1 and miRNA-16 in prostate cancer progression.

Finally, we investigated if there are biological experiments that validated our identification of the importance of the dysregulated protein complexes and miRNA modules in prostate cancer progression. Hudson *et al* [5] demonstrated that miRNA-1, involved in histone deacetylation, has a tumor suppression role and acts as a prognostic and diagnostic biomarker. miRNA-204 is another key regulator in prostate cancer progression as it regulates several genes, like MEIS1 and MEIS2, as a result of protein complex dysregulation [5]. Deregulation of Hox protein cofactors MEIS2, MEIS1 and Pbx1 are associated with cancer oncogenesis and tumor progression [44]. The role of MEIS2 and MEIS1 in low-grade prostate tumors suggests that they play a critical function in the formation of poor prognosis. Another key regulator that was investigated in prostate cancer is SMAD4, which is a putative suppressor of prostate tumor progression [45]. SMAD4 is downregulated in metastatic prostate cancer and is downregulated in 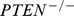. 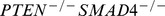 samples drive progression of PTEN-deficient prostate tumor to highly aggressive prostate cancer metastatic to lymphnode [45]. Another important pathway that is involved in prostate cancer progression is NF-kB mediated signaling. NF-kB inhibitors decrease AR expression levels, prostate-specific antigen secretion, and proliferation of prostate cancer cells in vitro [46]. Our results suggest that NF-kB pathway dysregulation is mediated by miRNAs.

### Conclusions

Investigating the functional role of miRNAs using a systems biology perspective helps us to understand the propagated influence of miRNAs on protein complexes in a particular biological context. Integrating the context (i.e. from gene expression data) of the protein targets of miRNAs is a promising step to identify miRNAs of high influence on gene expression of the target in the cell. This study provides a novel computational framework to identify dysregulated protein modules (complexes, pathways) influenced by miRNAs and find miRNA-target interactions consequently influenced by the dysregulated protein modules. The proposed framework reveals novel modules for further experimental design and investigations. Our proposed framework in this study identifies that protein complexes containing SMAD and HDAC proteins are the most influenced complexes by miRNAs. As a result of the dysregulation, other proteins like MEIS1, MEIS2, TPM1, and ZEB1, which are putative tumor suppressors, are affected. Finally, our developed framework can be generalized to find the influence of miRNAs on other curated gene sets data; TF-gene data for instance. The results of our framework reveal a previously unidentified layer of regulation that explains the dysregulation in biological pathways. Our results suggest that several cancer pathways (RAS, NF-kB) are under the control of miRNA-mediated regulation, and miRNA-1 and miRNA-16 are master regulators of miRNA-mediated regulation in prostate cancer.

## Supporting Information

File S1 **A compiled list of all supplementary figures.** Figure S1: Heatmap of SMAD6-HOXC8 complex,Figure S2: Heatmap of SMAD3-SMAD4-cSKI complex,Figure S3: Kaplan-Meier plot of the 23 proteins using Taylor data, Figure S4: Kaplan-Meier curves of the 23 protein across 1200 breast cancer samples,Figure S5: Kaplan-Meier plots of 21 miRNA from Taylor data,Figure S6: Kaplan-Meier plots of 21 miRNAs from Taylor data:three groups,Figure S7: Heatmap of 23 proteins in Taylor prostate data, Figure S8: Heatmap of 14 miRNAs(out of 21 miRNAs) using NCI-60 prostate data.
(DOCX)

Table S1 **A list of dysregulated protein complexes and their targeting miRNAs.** A list of the most significant protein complexes that were predicted to be dysregulated by miRNAs in prostate cancer. In each complex we show the CORUM ID, Complex Name, Protein members and the miRNAs targeting the genes encoding the proteins.
(PDF)

Table S2 **A list of miRNAs having role in prostate cancer based on literature.** This table lists the miRNAs that play a role in prostate cancer progression based on our analyzed expression data sets; either prostate cell lines or prostate tissue. It also lists the experimentally verified targets for some of them that we extracted from literature.
(PDF)

Table S3 **A list of miRNA-target interactions affected by the dysregulation of the dysregulated complexes.** This table shows the miRNA-target interactions dependent on the protein complexes we are interested in. We found that miRNA-1 and miRNA-16 interactions are significantly dependent on the protein RAF, which is part of the G-protein complex.
(XLSX)
