# Supplementary material for: Coordinate MicroRNA-Mediated Regulation of Protein Complexes in Prostate Cancer
Source: PLoS One. 2013 Dec 31;8(12):e84261. doi: 10.1371/journal.pone.0084261 (PMC3877262; doi:10.1371/journal.pone.0084261)
Supplement: File S1 — A compiled list of all supplementary figures. Figure S1: Heatmap of SMAD6-HOXC8 complex,Figure S2: Heatmap of SMAD3-SMAD4-cSKI complex,Figure S3: Kaplan-Meier plot of the 23 proteins using Taylor data, Figure S4: Kaplan-Meier curves of the 23 protein across 1200 breast cancer samples,Figure S5: Kaplan-Meier plots of 21 miRNA from Taylor data,Figure S6: Kaplan-Meier plots of 21 miRNAs from Taylor data:three groups,Figure S7: Heatmap of 23 proteins in Taylor prostate data, Figure S8: Heatmap of 14 miRNAs(out of 21 miRNAs) using NCI-60 prostate data. (DOCX) [file pone.0084261.s001.docx]

**Coordinate microRNA-mediated regulation of protein complexes in prostate cancer**

Mohammed Alshalalfa, Gary Bader, Tarek A.Bismar and Reda Alhajj

Supplementary figures (S1-S8)


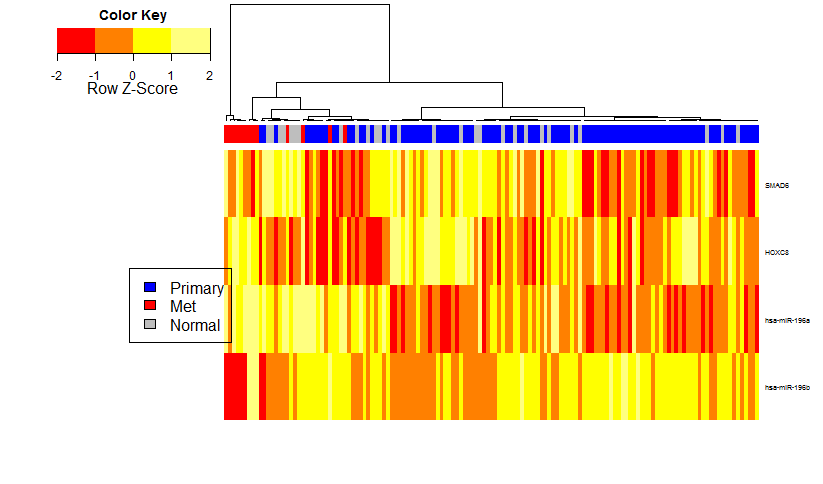


Figure S1: Heatmap of SMAD6-HOXC8 complex. This heatmap shows the heatmap of the members of SMAD6-HOXC8 complex and the miRNAs targeting them . We used Taylor data for the expression data.


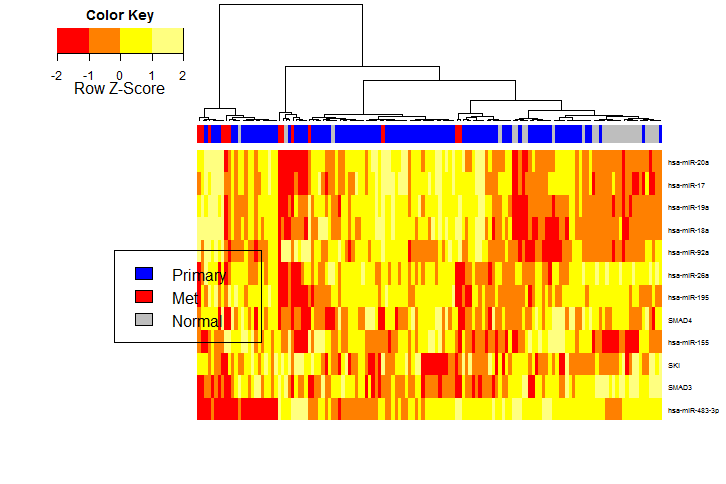


Figure S2: Heatmap of SMAD3-SMAD4-cSKI complex. This heatmap shows the heatmap of the members of SMAD3-SMAD4-cSKI complex and the miRNAs targeting them . We used Taylor data for the expression data.


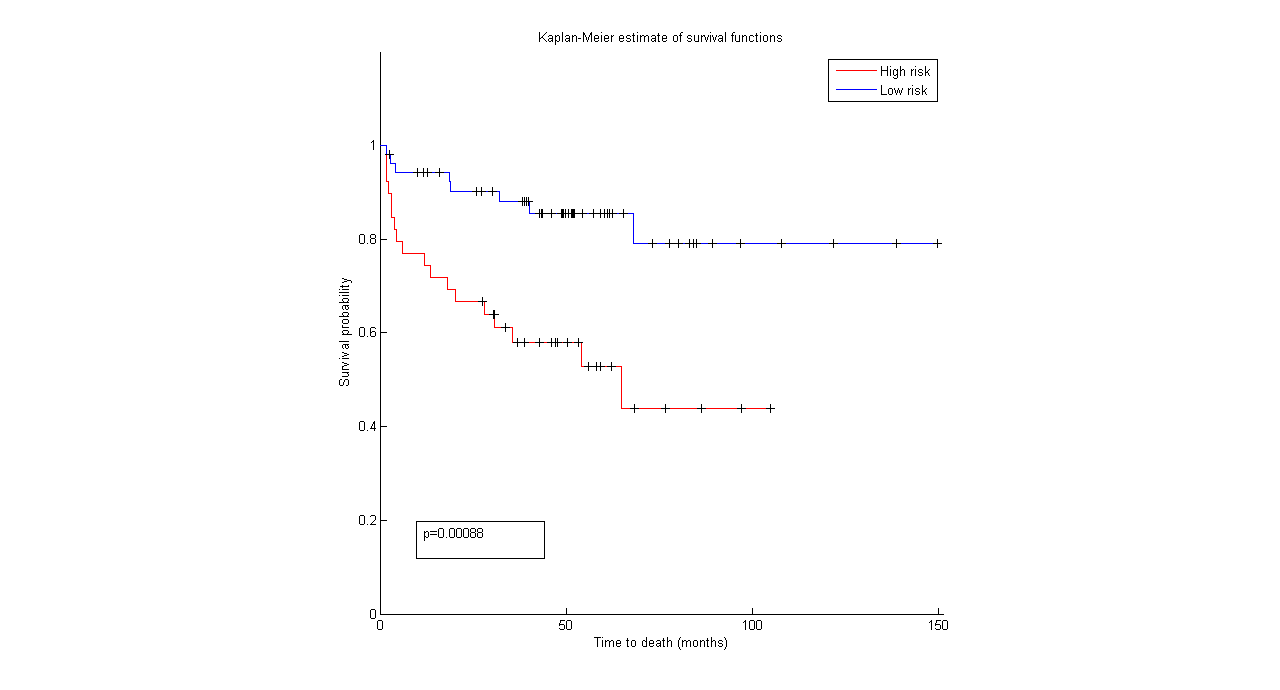


Figure S3: Kaplan-Meier plot of the 23 proteins using Taylor data

Here we further groups the samples across 23 proteins into 3 groups (Low risk, intermediate risk and high risk). Results revealed that low risk and high risk groups are significantly separated, and intermediate group is not well separated from the other two plots.


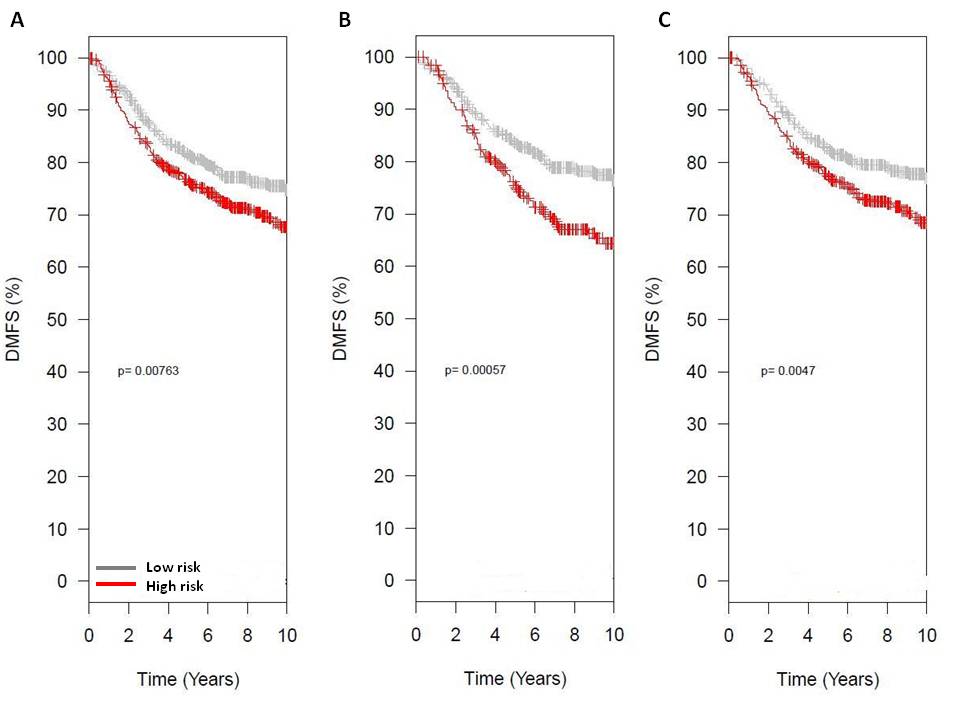


Figure S4: Kaplan-Meier curves of the 23 protein across 1200 breast cancer samples.

Figures are produced using GOBO online tool. A. shows that the 23 proteins are associated with breast cancer metastasis across all samples in the GOBO database. B. shows that the 23 proteins are associated with cancer metastasis in ER-positive subtype and C. shows that the 23 proteins are associated with metastasis with LN-negative breast cancer subtype.


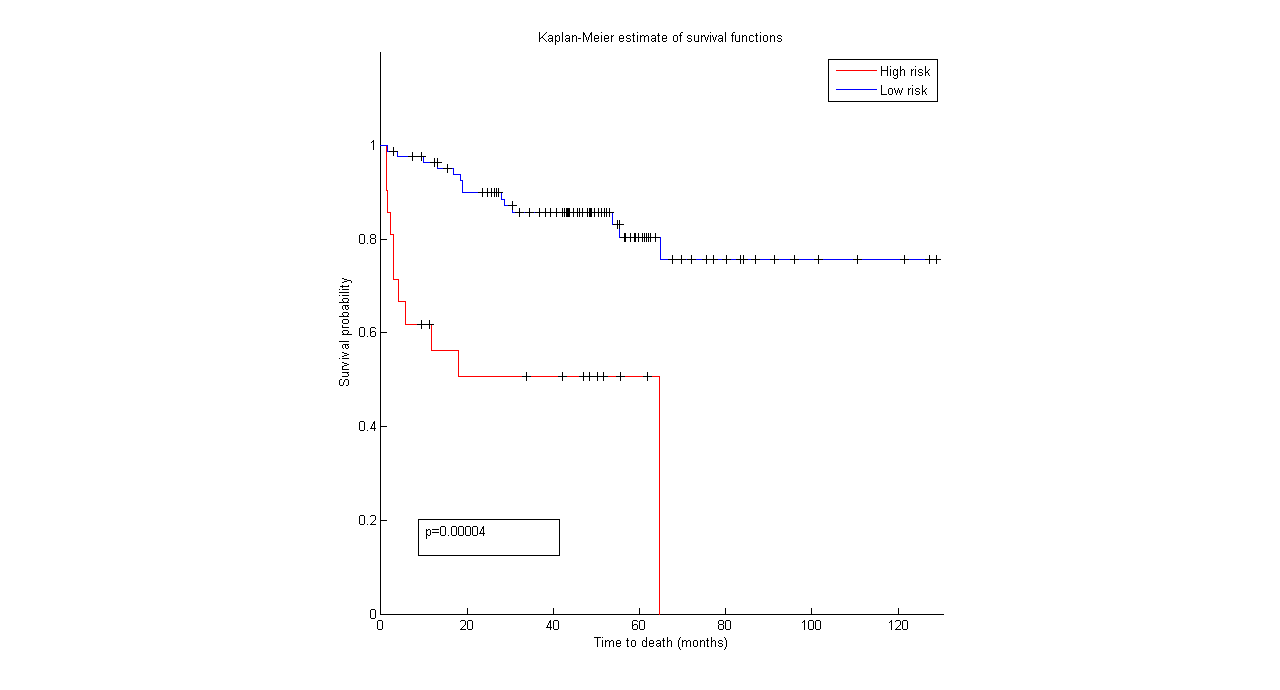


Figure S5: Kaplan-Meier plots of 21 miRNA from Taylor data

Expression of 21 miRNAs are extracted from Taylor data, then grouped into two groups. The resulted two groups are clinical distinct.


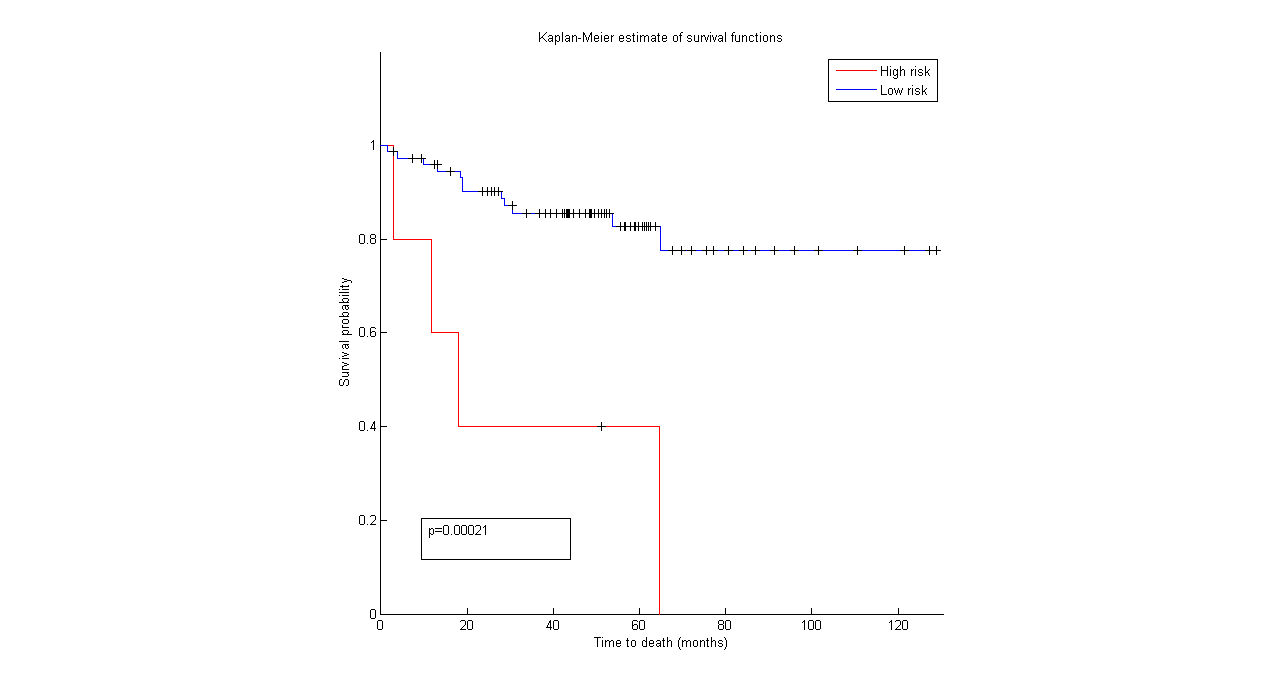


Figure S6: Kaplan-Meier plots of 21 miRNAs from Taylor data (three groups)

Using the expression of the 21 miRNAs from Taylor data and then grouping the samples into 3 groups revealed that the resulted groups harbor significant clinical information. Here we show the plots of the high risk vs low risk groups. The intermediate group was not significantly separated from the other two.


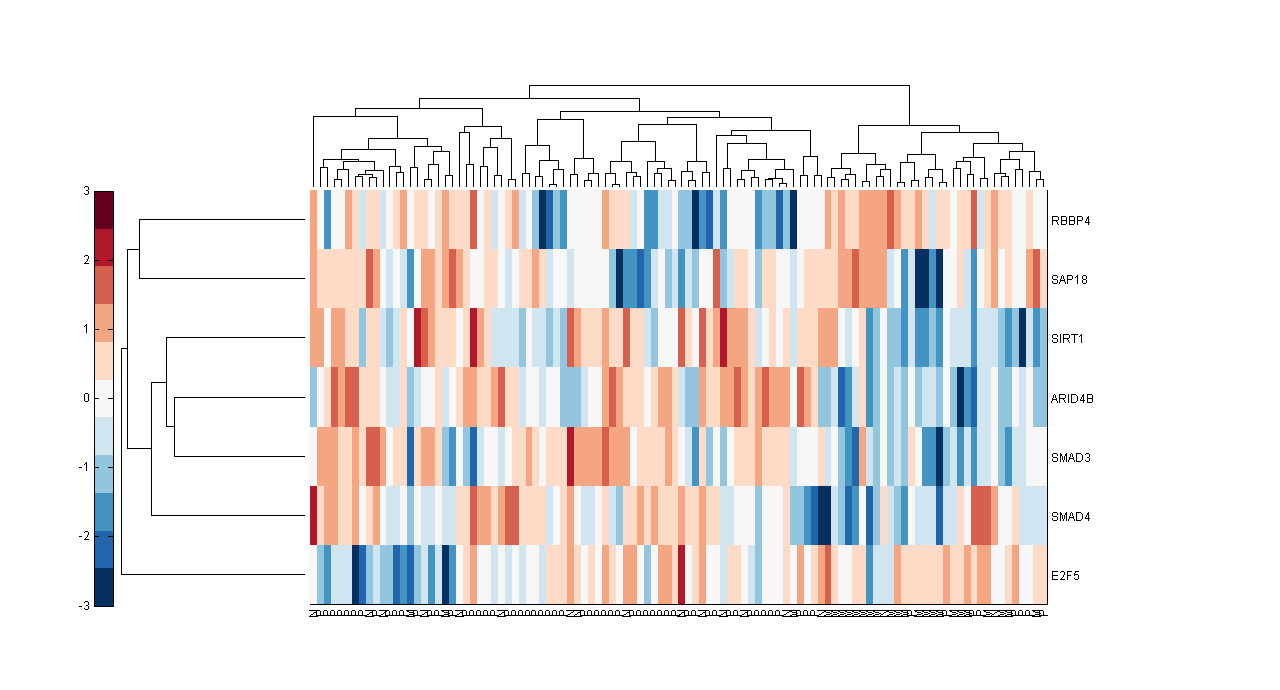
 Figure S7: Heatmap of 23 proteins in Taylor prostate data

We extracted expression of 7 proteins out of 23 from Taylor prostate data and then used hierarchical clustering to group samples. Results show that metastasis (right most) samples are clearly distinct from the normal and primary samples.


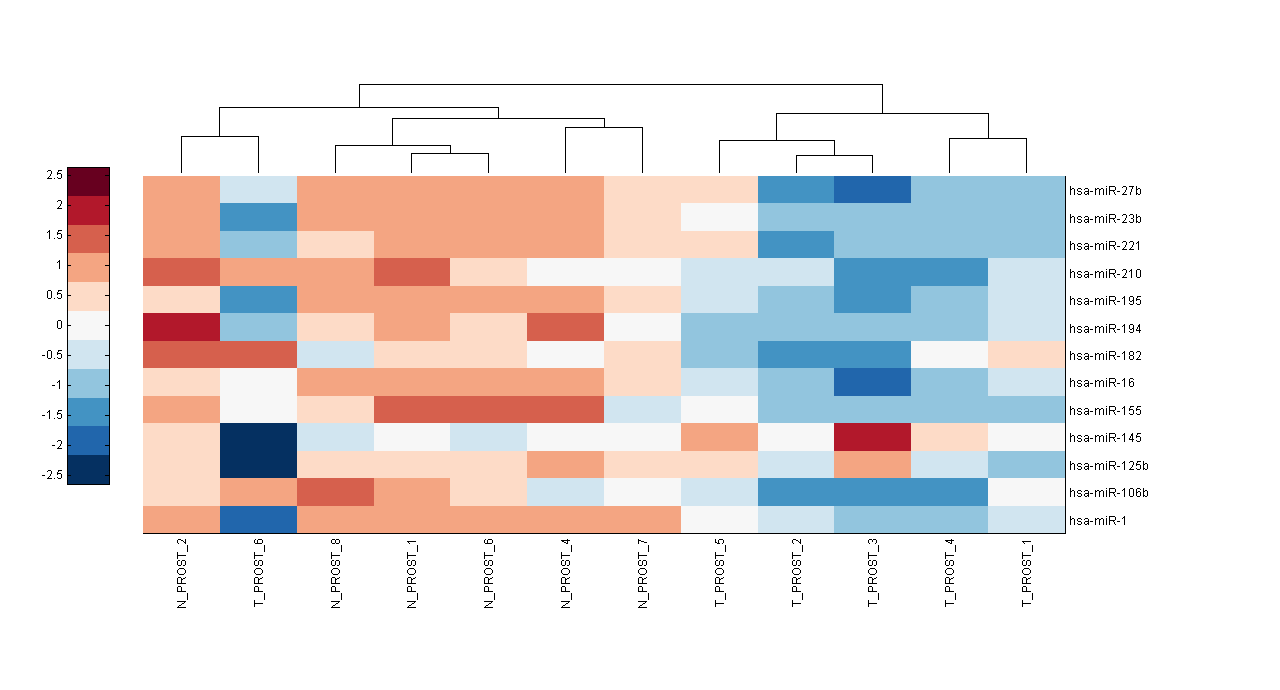


Figure S8: Heatmap of 14 miRNAs(out of 21 miRNAs) using NCI-60 prostate data

Expression of 14 miRNAs (out of 21 miRNA) are extracted from NCI-60 prostate data and then clustered using hierarchical clustering. Results show that the 14 miRNA are powerfull diagnostic markers that can distinguish tumor from normal samples.
