# Supplementary material for: Coordinate MicroRNA-Mediated Regulation of Protein Complexes in Prostate Cancer
Source: PLoS One. 2013 Dec 31;8(12):e84261. doi: 10.1371/journal.pone.0084261 (PMC3877262; doi:10.1371/journal.pone.0084261)
Supplement: Table S1 — A list of dysregulated protein complexes and their targeting miRNAs. A list of the most significant protein complexes that were predicted to be dysregulated by miRNAs in prostate cancer. In each complex we show the CORUM ID, Complex Name, Protein members and the miRNAs targeting the genes encoding the proteins. (PDF) [file pone.0084261.s002.pdf]

Coordinate microRNA-mediated regulation of protein complexes in prostate cancer

Mohammed alshalalfa, Gary Bader, Tarek Bismar, Reda Alhajj

Table S1: A list of the most significant protein complexes that were predicted to be dysregulated by miRNAs in prostate cancer. In each complex we show the CORUM ID, Complex Name, Protein members and the miRNAs targeting the genes encoding the proteins.

CORUM ID: 4089	
Complex Name: SMAD6-HOXC8 complex	
Complex protein members	miRNAs targeting protein members
HOXC8	hsa-miR-196
HOXC8	hsa-miR-196a
HOXC8	hsa-miR-196b
SMAD6	hsa-miR-520h

CORUM ID :3959	
Complex Name: SMAD3-SMAD4-cSKI	
Complex protein members	miRNAs targeting protein members
SKI	hsa-miR-155
SKI	hsa-miR-195
SMAD4	hsa-miR-17
SMAD4	hsa-miR-18a
SMAD4	hsa-miR-19a
SMAD4	hsa-miR-20a
SMAD4	hsa-miR-26a
SMAD4	hsa-miR-483-3p
SMAD4	hsa-miR-92a

CORUM ID: 3740	
Complex Name: SKI-SMAD3-SMAD4 pentameric complex	
Complex protein members	miRNAs targeting protein members
SKI	hsa-miR-155
SKI	hsa-miR-195
SMAD4	hsa-miR-17
SMAD4	hsa-miR-18a
SMAD4	hsa-miR-19a
SMAD4	hsa-miR-20a
SMAD4	hsa-miR-26a

SMAD4	hsa-miR-483-3p
SMAD4	hsa-miR-92a

CORUM ID: 2447	
Complex Name: ITGA9-ITGB1-ADAM12 complex	
Complex protein members	miRNAs targeting protein members
ITGB1	hsa-miR-124
ITGB1	hsa-miR-183
ITGA9	hsa-miR-194
ADAM12	hsa-miR-29b

CORUM ID: 1539	
Complex Name: G protein complex	
Complex protein members	miRNAs targeting protein members
RAF1	hsa-miR-125b
RAF1	hsa-miR-7

CORUM ID: 5589	
Complex Name: LINC complex	
Complex protein members	miRNAs targeting protein members
RBL1	hsa-miR-106b
RBL1	hsa-miR-17
RBL1	hsa-miR-20a

CORUM ID: 2590	
Complex Name: FOXO1-FHL2-SIRT1 complex	
Complex protein members	miRNAs targeting protein members
FOXO1	hsa-miR-153
FOXO1	hsa-miR-182
FOXO1	hsa-miR-183
FOXO1	hsa-miR-186
FOXO1	hsa-miR-27a
FOXO1	hsa-miR-9
FOXO1	hsa-miR-96
SIRT1	hsa-miR-132
SIRT1	hsa-miR-216a
SIRT1	hsa-miR-217
SIRT1	hsa-miR-34a

CORUM ID: 695	
Complex Name: SIN3A-HDAC complex	
Complex protein members	miRNAs targeting protein members
HDAC1	hsa-miR-410
HDAC2	hsa-miR-142-5p
SIN3A	hsa-miR-138
SIN3A	hsa-miR-144
SIN3A	hsa-miR-149
SIN3A	hsa-miR-183
SIN3A	hsa-miR-204
SIN3A	hsa-miR-330
SIN3A	hsa-miR-431
SIN3A	hsa-miR-493-5p
SIN3A	hsa-miR-9
SAP30	hsa-miR-30
RBBP7	hsa-miR-101
RBBP7	hsa-miR-144
RBBP7	hsa-miR-181
RBBP7	hsa-miR-186
RBBP7	hsa-miR-199a*
RBBP7	hsa-miR-20
RBBP7	hsa-miR-381
RBBP7	hsa-miR-539
RBBP7	hsa-miR-543
RBBP7	hsa-miR-544
RBBP7	hsa-miR-93
RBBP4	hsa-miR-133
RBBP4	hsa-miR-185
ARID4B	hsa-let-7g
ARID4B	hsa-let-7i
ARID4B	hsa-miR-101
ARID4B	hsa-miR-106b
ARID4B	hsa-miR-128
ARID4B	hsa-miR-128a
ARID4B	hsa-miR-128b
ARID4B	hsa-miR-130
ARID4B	hsa-miR-130a
ARID4B	hsa-miR-130b
ARID4B	hsa-miR-137
ARID4B	hsa-miR-142-5p
ARID4B	hsa-miR-144

ARID4B	hsa-miR-17-5p
ARID4B	hsa-miR-183
ARID4B	hsa-miR-19
ARID4B	hsa-miR-193a
ARID4B	hsa-miR-193b
ARID4B	hsa-miR-199a*
ARID4B	hsa-miR-19a
ARID4B	hsa-miR-19b
ARID4B	hsa-miR-20
ARID4B	hsa-miR-200b
ARID4B	hsa-miR-20a
ARID4B	hsa-miR-20b
ARID4B	hsa-miR-218
ARID4B	hsa-miR-30
ARID4B	hsa-miR-301
ARID4B	hsa-miR-302a
ARID4B	hsa-miR-302b*
ARID4B	hsa-miR-302c*
ARID4B	hsa-miR-30a-5p
ARID4B	hsa-miR-30b
ARID4B	hsa-miR-30c
ARID4B	hsa-miR-30d
ARID4B	hsa-miR-30e-5p
ARID4B	hsa-miR-328
ARID4B	hsa-miR-34
ARID4B	hsa-miR-34a
ARID4B	hsa-miR-34b
ARID4B	hsa-miR-34c
ARID4B	hsa-miR-370
ARID4B	hsa-miR-374
ARID4B	hsa-miR-377
ARID4B	hsa-miR-378
ARID4B	hsa-miR-381
ARID4B	hsa-miR-409-5p
ARID4B	hsa-miR-410
ARID4B	hsa-miR-452
ARID4B	hsa-miR-493-5p
ARID4B	hsa-miR-494
ARID4B	hsa-miR-495
ARID4B	hsa-miR-496
ARID4B	hsa-miR-505

ARID4B	hsa-miR-93
