# Supplementary material for: Coordinate MicroRNA-Mediated Regulation of Protein Complexes in Prostate Cancer
Source: PLoS One. 2013 Dec 31;8(12):e84261. doi: 10.1371/journal.pone.0084261 (PMC3877262; doi:10.1371/journal.pone.0084261)
Supplement: Table S2 — A list of miRNAs having role in prostate cancer based on literature. This table lists the miRNAs that play a role in prostate cancer progression based on our analyzed expression data sets; either prostate cell lines or prostate tissue. It also lists the experimentally verified targets for some of them that we extracted from literature. (PDF) [file pone.0084261.s003.pdf]

Coordinate microRNA-mediated regulation of protein complexes in prostate cancer

Mohammed alshalalfa, Gary Bader, Tarek Bismar, Reda Alhajj

Table S2: This table lists the miRNAs that play a role in prostate cancer progression based on our analyzed expression data sets; either prostate cell lines or prostate tissue. It also lists the experimentally verified targets for some of them that we extracted from literature.

miRNA	Known targets in prostate context (cell line or tissue)	Reference	Function	Experimentally verified target pathways
miRNA-221	$P27^{Kip1}$ (CDKN1B)	1,8	Oncogene	Development and metastasis of PCa, AR–mediated signaling
miRNA-222	$P27^{Kip1}$ (CDKN1B)	1,8	Oncogene	Development and metastasis of PCa, AR–mediated signaling
miRNA-21	MARCKS<br>PDCD4<br>TPM1<br>BMPRII	1,8<br>1,8<br>1,8<br>3,8	Oncogene	Development and metastasis of PCa , AR-mediate signaling
miRNA-125b	BAK1<br>EIF4EBP1	1,8<br>1	Oncogene	cell proliferation
miRNA-101	EZH2	1,3,8	Tumor suppressor	Cell growth and invasiveness
miRNA-126*	Prostein (SLC45A3)<br>EGFL7	1,8<br><br>2	Tumor suppressor	VEGF signaling and vessel development
miRNA-146a	ROCK1	1,3	Tumor suppressor	HA/Rock1 pathway
miRNA-330	E2F1	3	Tumor suppressor	AKT signaling
miRNA-200a	ZEB1<br>ZEB2	3<br>3	Tumor suppressor	epithelial to mesenchymal transition
miRNA-200b	ZEB1	3	Tumor	epithelial to

	ZEB2	3	suppressor	mesenchymal transition
miRNA-429		3	Tumor suppressor	epithelial to mesenchymal transition
miRNA-200c	ZEB1<br>ZEB2	3,8		
miRNA-141		3		
miRNA-34a	P53<br>CDK6<br>SIRT1	3,8<br>3,8<br>3,8	Tumor suppressor	AR, p53 pathway
miRNA-17-5p		3	Tumor suppressor	
miRNA-17-3p	VIM	3	Tumor suppressor	
miRNA-193b		8		
miRNA-92		3		
miRNA-106a		3,8		
miRNA-155		3,8		
miRNA-338		3		
miRNA-126		3		
miRNA-181b		3,8		
miRNA-181c		3		
miRNA-224		3,8		
miRNA-23b	c-MYC<br>GLS	3		
miRNA-100	SMARCA5<br>SMARCD1<br>mTOR	3,6		
miRNA-145	ICP4<br>MYO6	3,4,8		
miRNA-15a	BCL2<br>CCND1<br>WNT3A	3	Tumor suppressor	VEGF signaling
miRNA-16	BCL2<br>CCND1<br>WNT3A	3,8	Tumor suppressor	VEGF signaling
miRNA-23a	c-MYC<br>GLS	3	Tumor suppressor	
miRNA-449a	HDAC1	3	Tumor suppressor	
miRNA-331-3p	CDCA5<br>KIF23<br>ERBB2	3<br>3<br>3,8	Tumor suppressor	
miRNA-373	CD44	3	Tumor suppressor	

miRNA-520c	CD44	3	Tumor suppressor	
miRNA-205	ZEB2<br>ERBB3<br>PRKCE	3,8<br>3,8<br>3,8	Tumor suppressor	
miRNA-143	ERK5<br>MYO6<br>ICP4	3,8<br>8<br>8	Tumor suppressor	
miRNA-20a	E2F1<br>E2F2<br>E2F3	3<br>3<br>3	Oncogene	
miRNA-106b	E2F1<br>CDKN1A	3<br>3	Oncogene	
miRNA-32	Bim1<br>BCL2	3	Oncogene	
miRNA-218		3		
miRNA-let7c		3,8		
miRNA-1	HDAC4<br>XPO6<br>Notch3	4	Tumor suppressor	Inhibiting cell proliferation
miRNA-206	HDAC4	4		
miRNA-133a		4		
miRNA-182	SLC39A1	5		
miRNA-183		5		
miRNA-96		5		
miRNA-99a	SMARCA5<br>SMARCD1<br>mTOR(FRAP1)<br>KLK3	6<br>6<br>6		
miRNA-99b	SMARCA5<br>SMARCD1<br>mTOR (FRAP1)<br>KLK3	6		
miRNA-204		7		
miRNa-210		7		
miRNA-451		7		
mi-RNA425-5p		7		

*: indicates that the miRNA is predicted to have high influence on target partners based on miRNA-target influence network

Reference:

[1] Pang Y , Young CY and Yuan H, MicroRNAs and prostate cancer, Acta Biochim Biophys Sin, 2010, 42:363-369

[2] Nikolic I, Plate K, Schmidt M, EGFL7 meets miRNA-126: an angiogenesis alliance, Vascular Cell, 2010, 2:9

[3] Saini S, Majid S, and Dahiya R, Diet, microRNAs and prostate cancer, Pharm Res, 2010, 27:1014-1026

[4] Hudson PS, et al, MicroRNA-1 is a candidate tumor suppressor and prognostic marker in human prostate cancer, Nucleic Acids Research, 2011, 1-15

[5] Mihelich BL, et al, miR-183-96-182 cluster is overexpressed in prostate tissue and regulates zinc homeostasis in prostate cells, The J.of Biol Chem, 2011, 296:44503-44511

[6] Sun D, et al, miR-99 family of microRNAs suppresses the expression of prostate-specific antigen and prostate cancer cell proliferation, Cancer Research, 2011, 71:1313-1324

[7] Martens-Uzunova ES, et al, Diagnostic and prognostic signature from the small non-coding RNA transcriptome in prostate cancer, Oncogene, 2012, 31:978-991

[8] Xu J et al, Prioritizing candidate disease miRNAs by topological features in the miRNA target-dysregulated network: case study of prostate cancer, Molecular Cancer Therapeutics, 2011,10,1857-1866
